# Supplementary material for: An engineered biosensor enables dynamic aspartate measurements in living cells
Source: bioRxiv. 2023 Nov 28:2023.06.27.546775. Originally published 2023 Jun 27. Preprint. [Version 2] doi: 10.1101/2023.06.27.546775 (PMC10327124; doi:10.1101/2023.06.27.546775)
Supplement: Supplement 1 [file media-1.docx]

Supplementary information: Protein engineering and in vitro characterization.

**iAspSnFR2, annotated sequence**

Mutations conferring aspartate specificity (S27A and S72P) was inserted into a precursor of iGluSnFR3 (iGluSnFR3-pre) to obtain iAspSnFR2.

Numbering according to 2VHA.pdb (glutamate binding protein, purple) and 2B3P.pdb (superfolder GFP, orange). Differences between iGluSnFR3.v857, iGluSnFR3-pre and iAspSnFR2 are:

GltI: E25K in iGluSnFR3-pre/iAspSnFR2, E25D in iGluSnFR3.v857

GltII: S27A in iAspSnFR2, S27S in iGluSnFR3-pre/iGlu3SnFR3.v857

GltI: S72P in iAspSnFR2, S72S in iGluSnFR3-pre/iGluSnFR3.v857

GltI: A184V in iGluSnFR3-pre/iAspSnFR2, A184S in iGluSnFR3.v857

cpSFVenus: S147S in iGluSnFR3-pre/iAspSnFR2, S147N in iGluSnFR3.v857

cpSFVenus: N147I in iGluSnFR3-pre/iAspSnFR2, N147T in iGluSnFR3.v857

cpSFVenus: T228 in iGluSnFR3-pre/iAspSnFR2, G228 in iGluSnFR3.v857

cpSFVenus: L46 in iGluSnFR3-pre/iAspSnFR2, F46 in iGluSnFR3.v857

cpSFVenus: GYG chromophore in iGluSnFR3-pre/iAspSnFR2, SYG chromophore in iGluSnFR3.v857

1 2 3 4 5 6

0 0 0 0 0 0

MRSAAGSTLDKIAKNGVIVVGHRKSAVPFSYYDSQHKVVGYSQEYSNAIVEAVKKKLNK

1 1 1

7 8 9 0 1 2

0 0 0 0 0 0

PDLQVKLIPITPQNRIPLLQNGTYDFECGSTTNNVERQKQAAFSDTIFVVGTRLLTKKGG

1 1 1 1 1 1

3 4 5 6 7 8

0 0 0 0 0 0

DIKDFANLKDKAVVVTSGTTSEVLLNKLNEEQKMNMRIISAKDHGDSFRTLESGRAVAFM

1 2 2 2 2 2

9 0 1 2 3 4

0 0 0 0 0 0

MDDVLLAGERAKAKKPDDWEIVGKPQSQEAYGCMLRKDDPQFKKLMDDTIAQVQTSGEAE

2 1 1 1 1 1 1

5 4 5 6 7 8 9

0 7 3 3 3 3 3

KWFDKWFKNPILVSHIVYITADKQKNGIKANFKIHHNVEDGSVQLADHYQQNTPIGDGPV

2 2 2 2 2

0 1 2 3 3

3 3 3 3 8 1 9

LLPDNHYLSYQSVLSKDPNEKRDHMVLLEFVTATTNSLGMDELYKGGTGGSMSKGEELFT

1 2 3 4 5 666

9 9 9 9 9 567

GVVPILVELDGDVNGHKFSVRGEGEGDATNGKLTLKLICTTGKLPVPWPTLVTTLGYGVQ

1 1 1

7 8 9 0 1 2

9 9 9 9 9 9

CFARYPDHMKQHDFFKSAMPEGYVQERTISFKDDGTYKTRAEVKLEGDTLVNRIELKGID

1 1 2 2 2

3 4 6 7 7

9 6 0 0 9

FKEDGNILGHKLEYNFNNPLNMNFELSDEMKALFKEPNDKALKLQHHHHHH

**Pairwise sequence alignment of iAspSnFR (Hellweg et al., 2023) and iAspSnFR2 (this work)**

Pairwise sequence alignment using the GFP and GltI domains only. GltI domain in purple, GFP in orange. Mutations conferring aspartate specificity to the iGluSnFR3 precursor (S27A and S72P) marked by yellow background.

Difference between iAspSnFR and iAspSnFR2 indicated by colon. A total of 25 mismatches and 497/522 = 95.2% sequence identity.

**iAspSnFR 1 ---AAGSTLDKIAKNGVIVVGHRESSVPFSYYDNQQKVVGFSQDYSNAIV 47**

**||||||||||||||||||||:|:|||||||:|:||||:||:||||||**

**iAspSnFR2 1 MRSAAGSTLDKIAKNGVIVVGHRKSAVPFSYYDSQHKVVGYSQEYSNAIV 50**

**iAspSnFR 48 EAVKKKLNKPDLQVKLIPITAQNRIPLLQNGTFDFECGSTDNNVERQKQA 97**

**||||||||||||||||||||:|||||||||||:|||||||:|||||||||**

**iAspSnFR2 51 EAVKKKLNKPDLQVKLIPITPQNRIPLLQNGTYDFECGSTTNNVERQKQA 100**

**iAspSnFR 98 AFSDTIFVVTTRLLTKKGGDIKDFANLKDKAVVVTSGTTSEVLLNKLNEE 147**

**|||||||||:||||||||||||||||||||||||||||||||||||||||**

**iAspSnFR2 101 AFSDTIFVVGTRLLTKKGGDIKDFANLKDKAVVVTSGTTSEVLLNKLNEE 150**

**iAspSnFR 148 QKMNMRIISAKDHGDSFRTLESGRAVAFMMDDVLLAGERARAKKPDNWEI 197**

**||||||||||||||||||||||||||||||||||||||||:|||||:|||**

**iAspSnFR2 151 QKMNMRIISAKDHGDSFRTLESGRAVAFMMDDVLLAGERAKAKKPDDWEI 200**

**iAspSnFR 198 VGKPQSQEAWGCMLRKDDPQFKKLMDDTIAQVRTSGEAEKWFDKWFKNPI 247**

**|||||||||:||||||||||||||||||||||:|||||||||||||||||**

**iAspSnFR2 201 VGKPQSQEAYGCMLRKDDPQFKKLMDDTIAQVQTSGEAEKWFDKWFKNPI 250**

**iAspSnFR 248 LVSHNVYITADKQKNGIKANFKIRHNVEDGSVQLADHYQQNTPIGDGPVL 297**

**||||:||||||||||||||||||:||||||||||||||||||||||||||**

**iAspSnFR2 251 LVSHIVYITADKQKNGIKANFKIHHNVEDGSVQLADHYQQNTPIGDGPVL 300**

**iAspSnFR 298 LPDNHYLSTQSVLSKDPNEKRDHMVLLEFVTAAGITLGMDELYKGGTGGS 347**

**||||||||:|||||||||||||||||||||||::::||||||||||||||**

**iAspSnFR2 301 LPDNHYLSYQSVLSKDPNEKRDHMVLLEFVTATTNSLGMDELYKGGTGGS 350**

**iAspSnFR 348 MSKGEELFTGVVPILVELDGDVNGHKFSVRGEGEGDATNGKLTLKFICTT 397**

**|||||||||||||||||||||||||||||||||||||||||||||:||||**

**iAspSnFR2 351 MSKGEELFTGVVPILVELDGDVNGHKFSVRGEGEGDATNGKLTLKLICTT 400**

**iAspSnFR 398 GKLPVPWPTLVTTLTYGVQCFSRYPDHMKQHDFFKSAMPEGYVQERTISF 447**

**||||||||||||||:||||||:||||||||||||||||||||||||||||**

**iAspSnFR2 401 GKLPVPWPTLVTTLGYGVQCFARYPDHMKQHDFFKSAMPEGYVQERTISF 450**

**iAspSnFR 448 KDDGTYKTRAEVKFEGDTLVNRIELKGIDFKEDGNILGHKLEYNFNNPLN 497**

**|||||||||||||:||||||||||||||||||||||||||||||||||||**

**iAspSnFR2 451 KDDGTYKTRAEVKLEGDTLVNRIELKGIDFKEDGNILGHKLEYNFNNPLN 500**

**iAspSnFR 498 MNFELSDEMKALFKEPNDKALK 519**

**||||||||||||||||||||||**

**iAspSnFR2 501 MNFELSDEMKALFKEPNDKALK 522**
