# Supplementary material for: An engineered biosensor enables dynamic aspartate measurements in living cells
Source: bioRxiv. 2023 Jun 27:2023.06.27.546775. Preprint. [Version 1] doi: 10.1101/2023.06.27.546775 (PMC10327124; doi:10.1101/2023.06.27.546775)
Supplement: Supplementary file 1 [file NIHPP2023.06.27.546775v1-supplement-1.pdf]

bioRxiv preprint doi: https://doi.org/10.1101/2023.06.27.546775; this version posted June 27, 2023. The copyright holder for this preprint (which was not certified by peer review) is the author/funder, who has granted bioRxiv a license to display the preprint in perpetuity. It is made available under aCC-BY 4.0 International license.

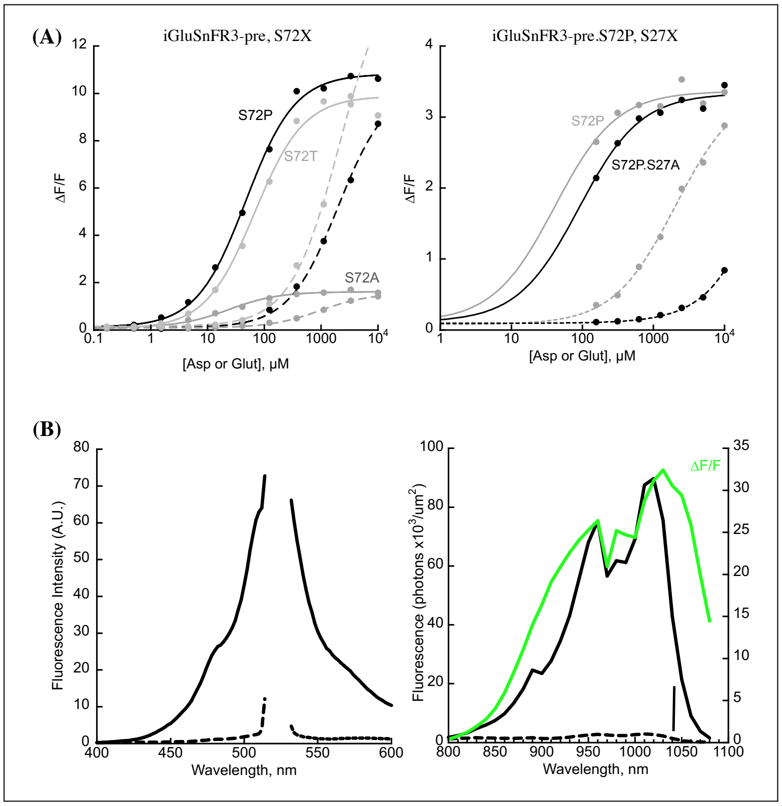

549 **Figure 1—figure supplement 1.** (A) Switching specificity of the iGluSnFR3 precursor from glutamate to aspartate using S72X library (left) and S72P, S27X library (right). Titrations with aspartate (solid lines) and glutamate (dashed lines) in bacterial lysate. (B) Excitation and emission spectra of jAspSnFR3-mRuby3. Left, 1-photon spectra. Excitation wavelength was varied from 400 nm to 520 nm (7.5 nm bandpass) while observing emission at 535 nm (10 nm bandpass). Emission wavelength was varied from 535 nm to 600 nm (10 nm bandpass) while exciting at 510 nm (7.5 nm bandpass). Fluorescence was measured both in the absence (dashed lines) and presence of 10 mM aspartate (solid lines). Right, 2-photon cross-sections, also ± 10 mM aspartate, with an overlay of calculated F/F (green). Vertical bar indicates 1040 nm.

bioRxiv preprint doi: https://doi.org/10.1101/2023.06.27.546775; this version posted June 27, 2023. The copyright holder for this preprint (which was not certified by peer review) is the author/funder, who has granted bioRxiv a license to display the preprint in perpetuity. It is made available under aCC-BY 4.0 International license.

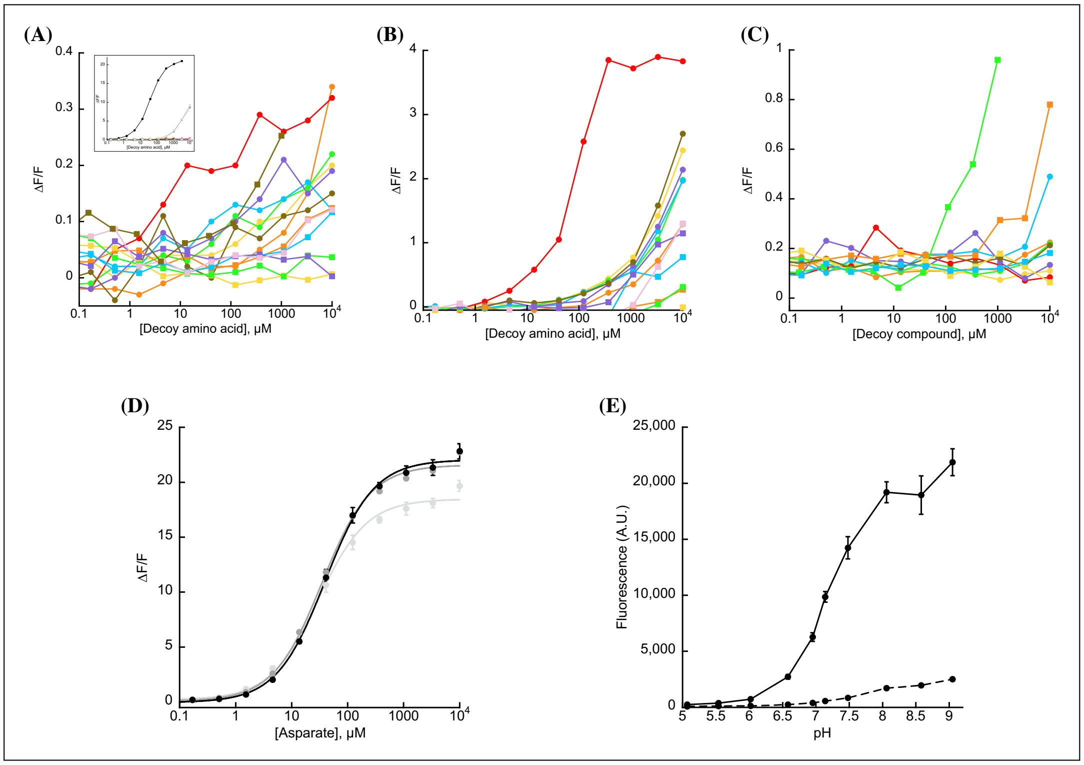

Figure 1—figure supplement 2. (A) jAspSnFR3-mRuby3 does not appreciably change its green fluorescence in response to other amino acids (alanine, phenylalanine, glycine, histidine (red line), isoleucine, leucine, methionine, proline, glutamine, arginine, serine, threonine, valine, or tryptophan). Insert with aspartate in black and glutamate/asparagine in grey for comparison. Ex. 485 nm (20 nm bandpass), Em. 535 nm (20 nm bandpass), 0.2 µM purified protein in PBS. (B) jAspSnFR3-mRuby3 shows increased red fluorescence at millimolar concentrations of all amino acids, with apparent responses to histidine at 100 µM (red trace). Ex. 587 nm (20 nm bandpass), Em. 662 nm (20 nm bandpass). (C) jAspSnFR3-mRuby3 does not respond to other decoys: citrate, lactate, pyruvate, malate, alpha-ketoglutarate, cis-aconitate, succinate, fumarate, or oxaloacetate (orange squares); nor to relevant pharmacological treatments: rotenone (green squares) or metformin. The small increase in fluorescence from rotenone is likely due to the scattering of a visibly turbid solution; rotenone has very low solubility in water. Ex. 485 nm (20 nm bandpass), Em. 535 nm (20 nm bandpass). (D) jAspSnFR3-mRuby3 is not adversely affected by temperature. Fluorescence as a function of aspartate titration at 23°C (light grey), 30°C (medium grey), and 37°C (black). Error bars are standard deviation of three technical replicates. (E) pH sensitivity of jAspSnFR3-mRuby3 (green component). Ex 485 nm (5 nm bp), Em 515 nm (10 nm bp). Error bars are standard deviation of 5 technical replicates. Solid line is with 3 mM aspartate, dashed line is without aspartate.

bioRxiv preprint doi: https://doi.org/10.1101/2023.06.27.546775; this version posted June 27, 2023. The copyright holder for this preprint (which was not certified by peer review) is the author/funder, who has granted bioRxiv a license to display the preprint in perpetuity. It is made available under aCC-BY 4.0 International license.

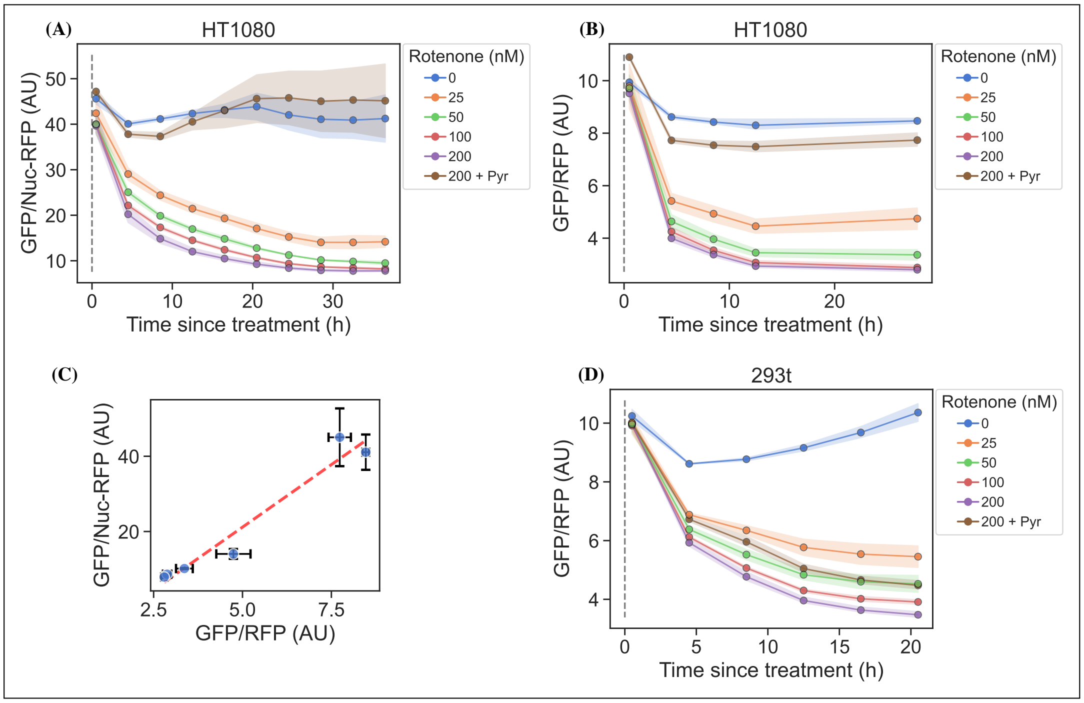

Figure 2—figure supplement 1. jAspSnFR3 temporal response after rotenone treatment. RFP normalized jAspSnFR3 signal change over time following rotenone treatment of live cells. Related to **Figure 2**, panel B; however, these experiments are not normalized to a pre-treatment scan. Grey dashed lines indicate the time of treatment and the first scan occurs 30 min after this. (A) HT1080 cells using nuclear RFP to normalize the jAspSnFR3 signal. (B) HT1080 cells using an RFP fused to jAspSnFR3 (jAspSnFR3-mRuby3) for normalization. (C) Comparison between the steady-state signal of (A) and (B) with a linear regression shown as a red dashed line to show that nuclear RFP and RFP fusion normalizations are equivalent. (D) HEK293t cells using an RFP fused to jAspSnFR3 for normalization. For plots (A), (B) and (D) markers indicate the average using available well replicates and are superimposed on a bootstrapped 95% confidence interval colored using the same color code as the markers. For plot (C) markers indicate the average using available well replicates and errorbars are drawn as +/- the standard deviation of the replicates. AU, arbitrary unit.

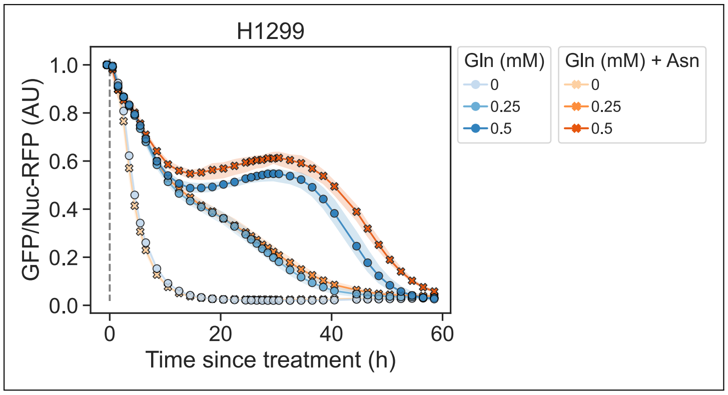

Figure 2—figure supplement 2. Aspartate depletes when glutamine is limiting. RFP normalized jAspSnFR3 signal change over time following glutamine depletion in H1299 cells. Identical to **Figure 2**, panel F but with fewer glutamine concentrations and more well replicates. AU, arbitrary unit.

bioRxiv preprint doi: https://doi.org/10.1101/2023.06.27.546775; this version posted June 27, 2023. The copyright holder for this preprint
(which was not certified by peer review) is the author/funder, who has granted bioRxiv a license to display the preprint in perpetuity. It is made
available under aCC-BY 4.0 International license.

553

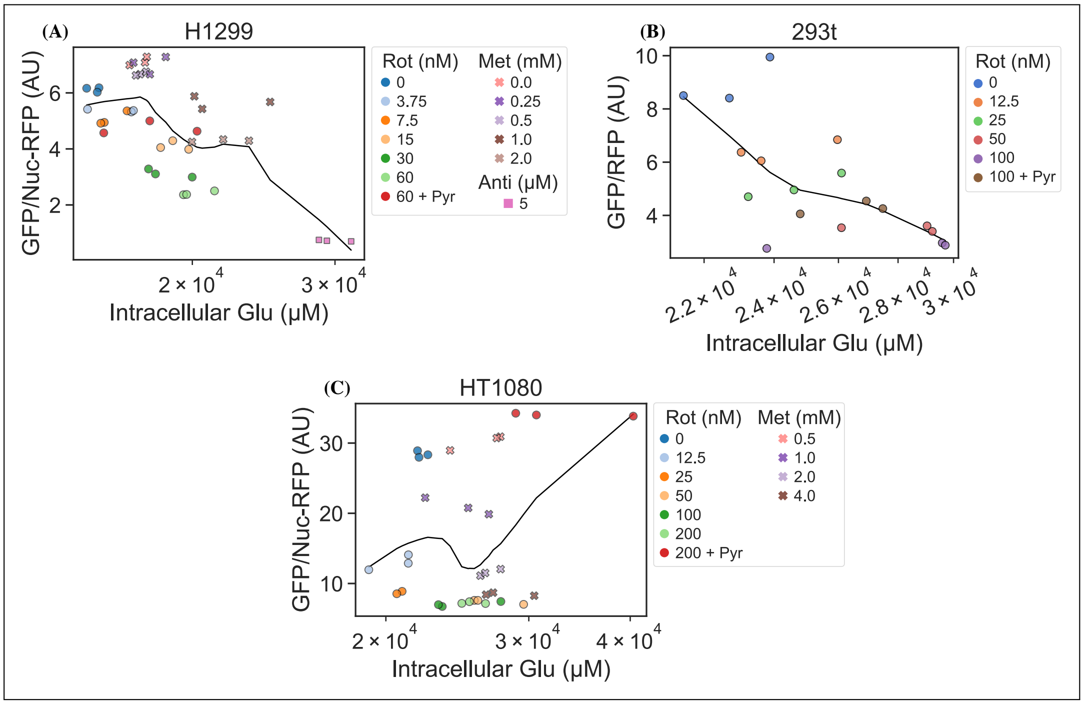

Figure 3—figure supplement 1. RFP normalized jAspSnFR3 signal, following various perturbations to live cells, is not correlated with the LCMS measured intracellular glutamate concentration. Datapoints are fitted to a local linear regression, shown by the black line, otherwise, these plots are identical to those in **Figure 3**. AU, arbitrary unit.
